# Supplementary material for: Accelerated pseudogenization on the neo-X chromosome in Drosophila miranda
Source: Nat Commun. 2016 Nov 29;7:13659. doi: 10.1038/ncomms13659 (PMC5141340; doi:10.1038/ncomms13659)
Supplement: Supplementary Information — Supplementary Figures 1 - 10, Supplementary Tables 1 - 11, Supplementary Methods and Supplementary References [file ncomms13659-s1.pdf]

SUPPLEMENTARY FIGURES

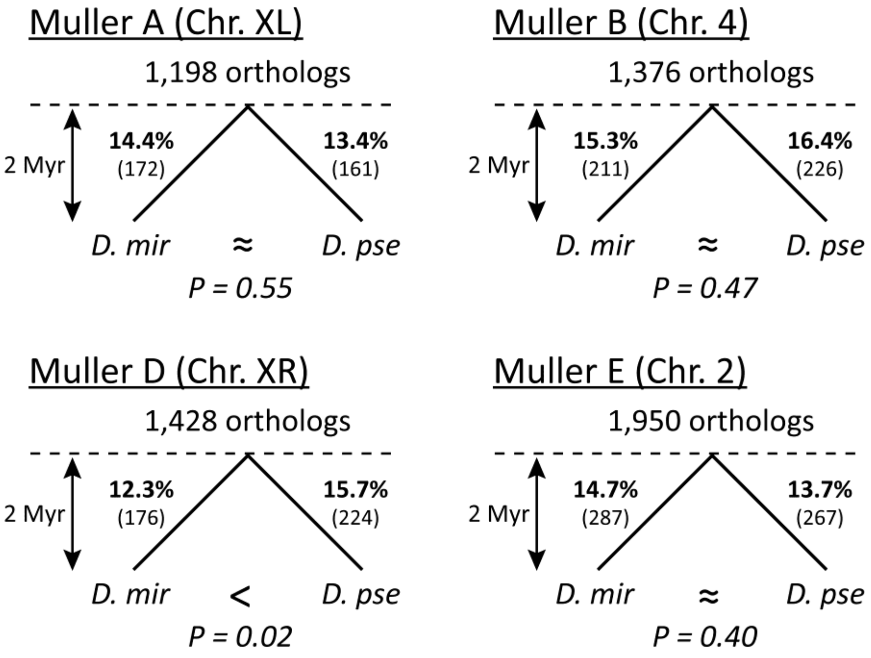

Supplementary Figure 1: Proportions of genes that became pseudogenized on each chromosome (i.e., Muller element) in the *D. miranda* and *D. pseudoobscura* lineages. Statistical significance of the difference in pseudogenization rate between the linages (i.e., *P* value under the null hypothesis of equal pseudogenization rates in the lineages) was examined by the χ^2 test. The number of orthologs used for the analysis is shown above the tree. The numbers in parentheses are the numbers of genes that were pseudogenized in each lineage. Genes on the Muller element F (chromosome 5) was unable to be analyzed because the chromosome assembly has not yet been available in *D. pseudoobscura*.

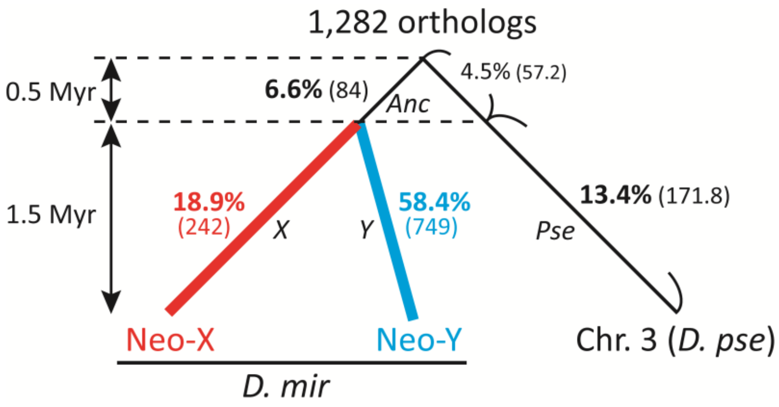

Supplementary Figure 2: Proportions of genes that became pseudogenized in each evolutionary lineage. Pseudogenization events in the lineage leading to *D. pseudoobscura* were split into two under the assumptions that neo-sex chromosomes emerged 1.5 Mya and a constant rate of pseudogenization in the lineage. Branch names that correspond to those in Supplementary Tables 8 and 9 are as follows: *Anc*, the ancestral branch before separating the neo-X and neo-Y; *X*, the neo-X branch; *Y*, the neo-Y branch; and *Pse*, the *D. pseudoobscura* branch after splitting from *D. miranda*.

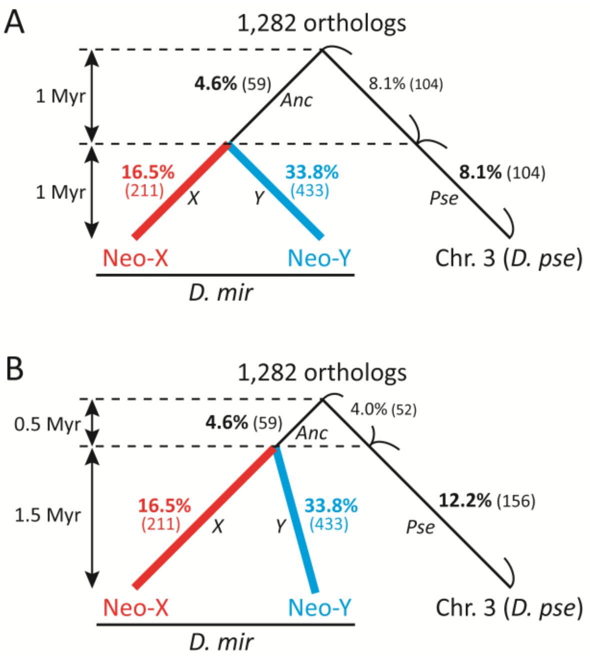

C

	P-value	
	1 Mya	1.5 Mya
X vs. *Y*	2.2×10^{-18}	2.2×10^{-18}
X vs. *Pse*	1.7×10^{-9}	4.1×10^{-3}
X vs. *Anc*	2.2×10^{-20}	0.08
Y vs. *Pse*	9.5×10^{-46}	3.6×10^{-30}
Y vs. *Anc*	8.7×10^{-64}	3.6×10^{-25}

Supplementary Figure 3: Proportions of genes that became pseudogenized in each evolutionary lineage, when the expression level of genes predicted solely by the genome sequence was also analyzed. (A) Proportions and numbers (in parentheses) of pseudogenization events under the assumption that neo-sex chromosomes emerged 1 Mya. Branch names are as follows: *Anc*, the ancestral branch before separating the neo-X and neo-Y; *X*, the neo-X branch; *Y*, the neo-Y branch; and *Pse*, the *D. pseudoobscura* branch after splitting from *D. miranda*. (B) Proportions and numbers (in parentheses) of pseudogenization events under the assumption that neo-sex chromosomes emerged 1.5 Mya. (C) Statistical significance by the χ^2 test under the null hypothesis that the rate of pseudogenization is the same between branches. Tests were conducted based on the two different emergence times of the neo-sex chromosomes. Branch names correspond to those in A and B. In this analysis, an expressed gene was defined as the gene that was expressed in at least one of the tissues examined with the FPKM value of ≥1.

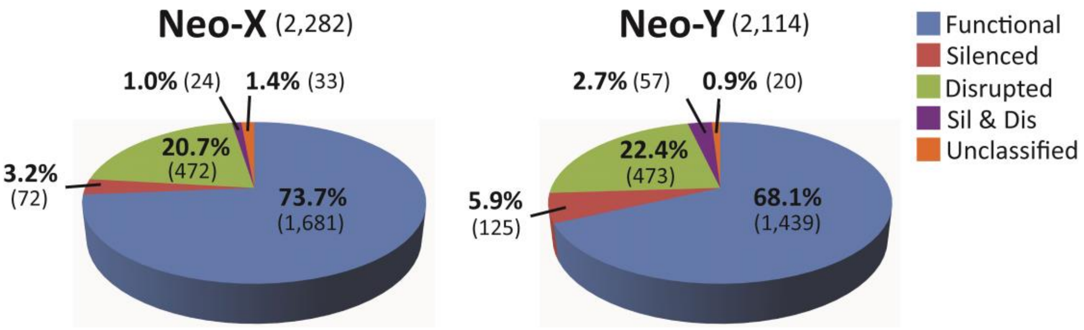

Supplementary Figure 4: Classification of neo-X and neo-Y genes, when the expression level of genes annotated solely by the genome sequence was also considered to determine the functionality of genes. Blue, red, green, purple, and orange colors mean functional, silenced, disrupted, silenced-and-disrupted, and unclassified genes, respectively. Silenced, disrupted, and silenced-and-disrupted genes were regarded as pseudogenes. The numbers in parentheses are the numbers of genes in each category. In this analysis, an expressed gene was defined as the gene that was expressed in at least one of the tissues examined with the FPKM value of ≥1.

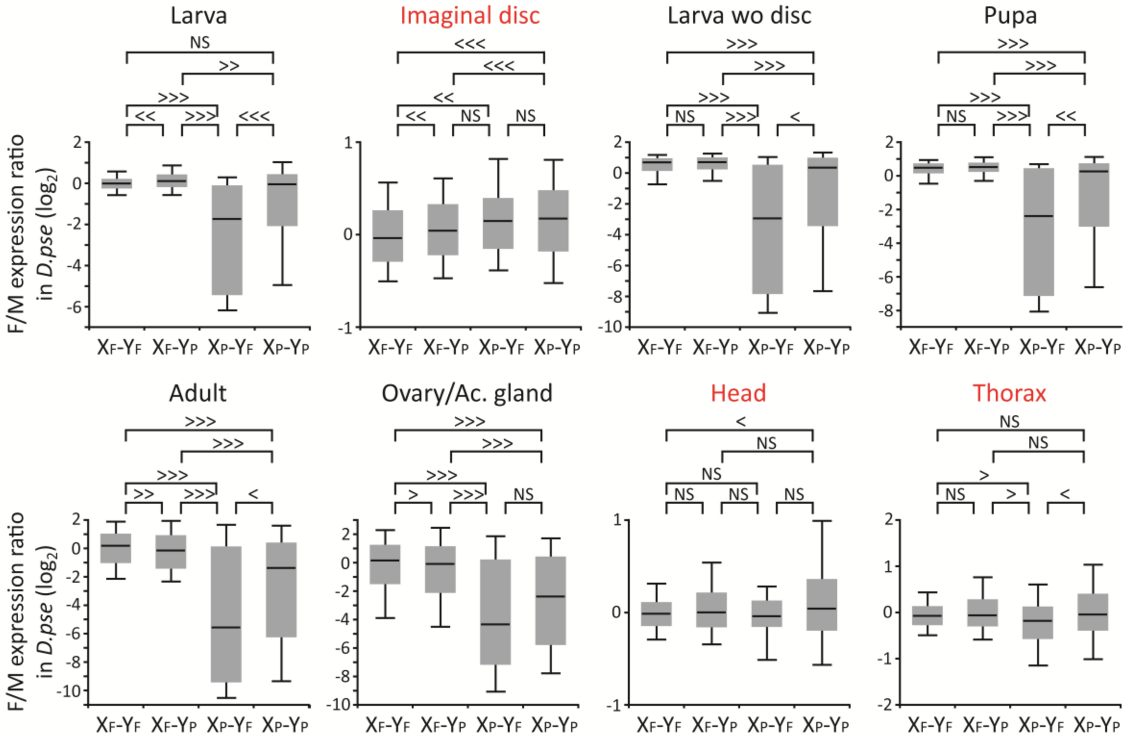

Supplementary Figure 5: Ratios of female to male gene expression in several tissues of *D. pseudoobscura*. The 1,672 *D. pseudoobscura* expressed genes whose orthologs are present in *D. obscura* and have not experienced any inter-chromosomal translocation after splitting from *D. obscura* were analyzed. The lines in the boxes represent medians, 50% of values are included in the boxes, and 80% of values are included within the bars. Statistical significance was calculated based on a Monte Carlo simulation with 1,000 bootstrap replicates: >>> or <<<, $P < 0.001$; >> or <<, $P < 0.01$; > or <, $P < 0.05$; NS, $P \geq 0.05$. Larva, larval whole bodies; Imaginal disc, larval imaginal discs except for genital discs; Larva wo disc, larval bodies without imaginal discs but containing genital discs; Pupa, pupal whole bodies; Adult, adult whole bodies; Ovary/Ac. Gland, ovaries in females and accessory glands in males; Head, adult heads; and Thorax, adult thoraxes. Tissues in red are the tissues that do not contain gonads or genital discs (i.e., only somatic tissues).

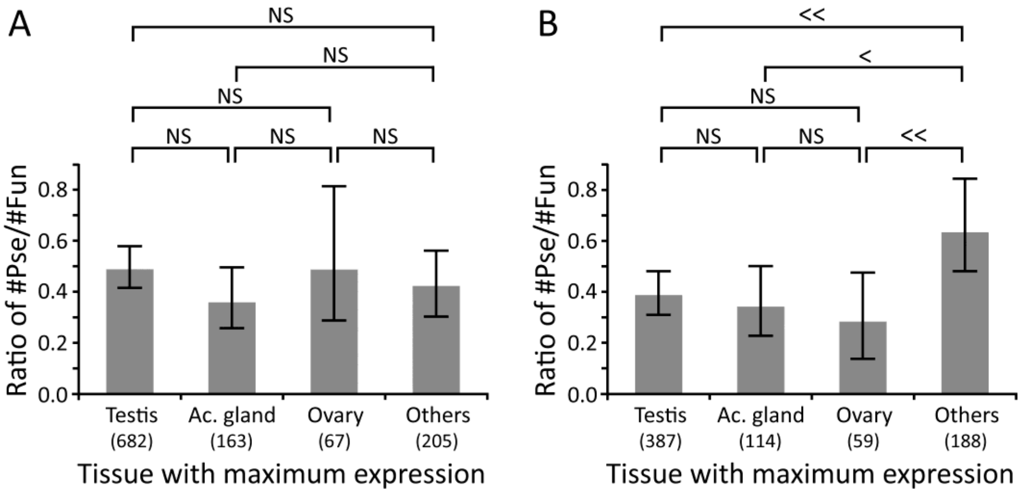

Supplementary Figure 6: Ratio of the number of pseudogenes to the number of functional genes depending on tissues with maximum expression (cFPKM, see Supplementary Methods for details). (A) Muller elements B and E (i.e., autosomes in both *D. miranda* and *D. pseudoobscura*). (B) Muller elements A and D (i.e., X chromosomes in both species). Error bars indicate the 95% confidence interval based on a Monte Carlo simulation with 1,000 bootstrap replicates. Numbers in parentheses are the numbers of genes with maximum expression in the respective tissues. Statistical significance was calculated based on a Monte Carlo simulation with 1,000 bootstrap replicates: >>> or <<<, $P < 0.001$; <<, $P < 0.01$; <, $P < 0.05$; NS, $P \geq 0.05$. In this analysis, only genes for which a cFPKM in a tissue was at least 2-fold compared with that in any of other tissues examined were considered to remove the genes for which cFPKMs were similar in multiple tissues.

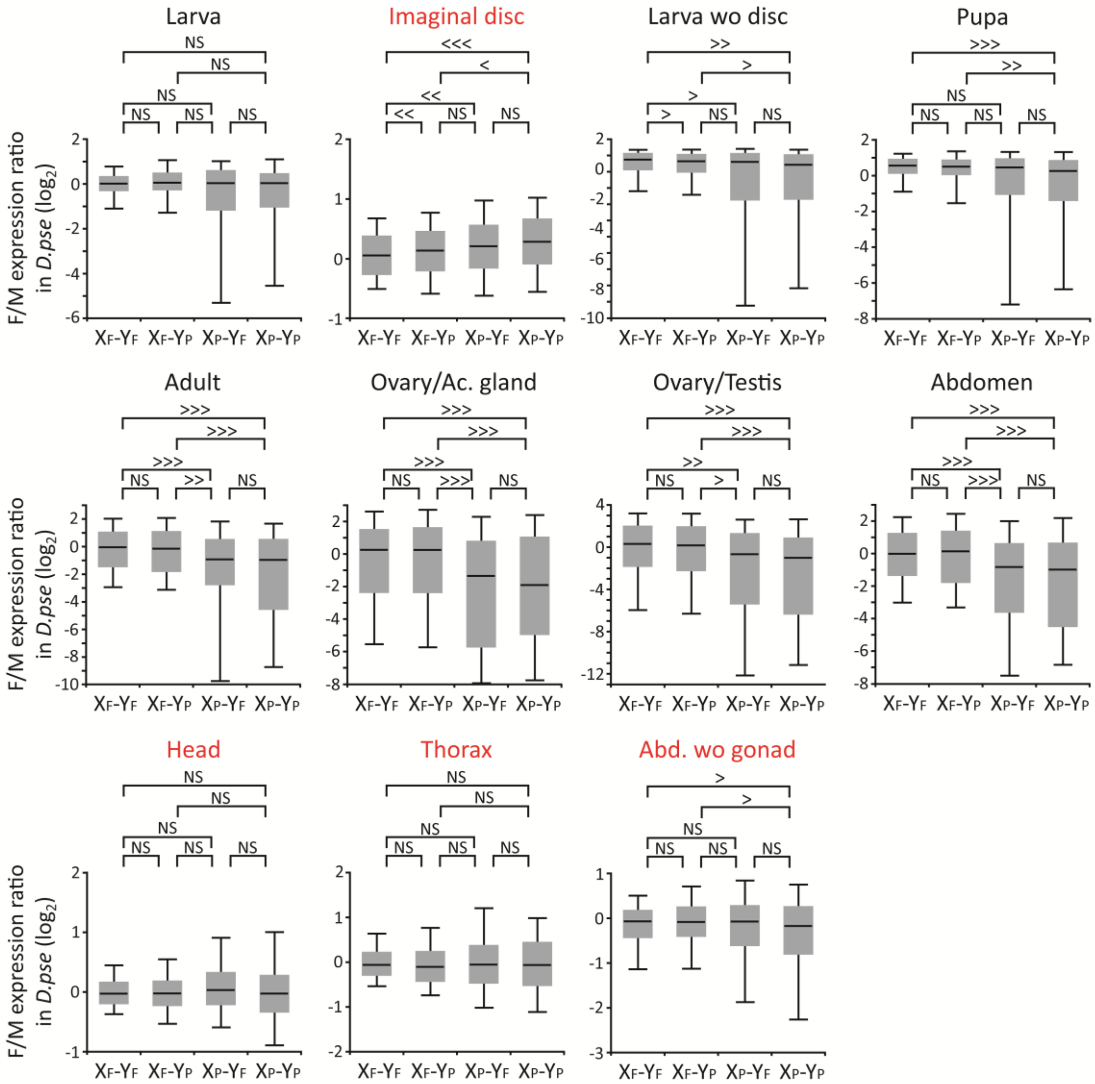

Supplementary Figure 7: Ratios of female to male gene expression in several tissues of *D. pseudoobscura*. In this analysis, the 1,722 *D. pseudoobscura* genes on chromosome 3 (i.e., Muller element 3) whose orthologs are present in *D. obscura* and have not experienced any inter-chromosomal translocation after splitting from *D. obscura* were analyzed. Here, the genes that were solely predicted by the genome sequence were also considered to determine the functionality of genes. In this analysis, an expressed gene was the gene with the FPKM value of ≥1 in at least one tissue. The lines in the boxes represent medians, 50% of values are included in the boxes, and 80% of values are included within the bars. Statistical significance was calculated

based on a Monte Carlo simulation with 1,000 bootstrap replicates: >>> or <<<, $P < 0.001$; >> or <<, $P < 0.01$; > or <, $P < 0.05$; NS, $P \geq 0.05$. Larva, larval whole bodies; Imaginal disc, larval imaginal discs except for genital discs; Larva wo disc, larval bodies without imaginal discs but containing genital discs; Pupa, pupal whole bodies; Adult, adult whole bodies; Ovary/Ac. Gland, ovaries in females and accessory glands in males; Ovary/Testis, ovaries in females and testes in males; Abdomen, abdomens; Head, adult heads; Thorax, adult thoraxes; and Abd. wo gonad, abdomens after removing gonads. Tissues in red are the tissues that do not contain gonads or genital discs (i.e., only somatic tissues).

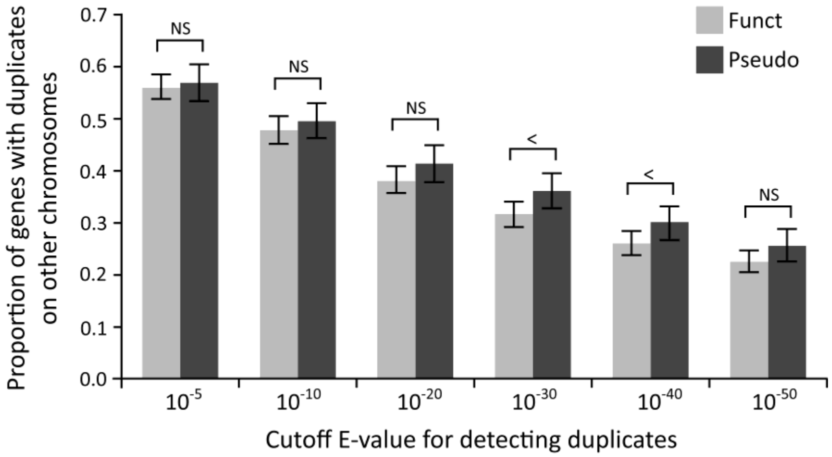

Supplementary Figure 8: Proportions of neo-X-linked genes that have duplicate genes on other chromosomes. Different E-value cutoffs were applied to detect duplicate genes. Light gray bars represent the proportion of functional neo-X-linked genes with duplicates on other chromosomes, whereas dark gray bars indicate the proportion of neo-X-linked pseudogenes. Statistical significance was calculated based on a Monte Carlo simulation with 1,000 bootstrap replicates: >>> or <<<, $P < 0.001$; <<, $P < 0.01$; <, $P < 0.05$; NS, $P \geq 0.05$.

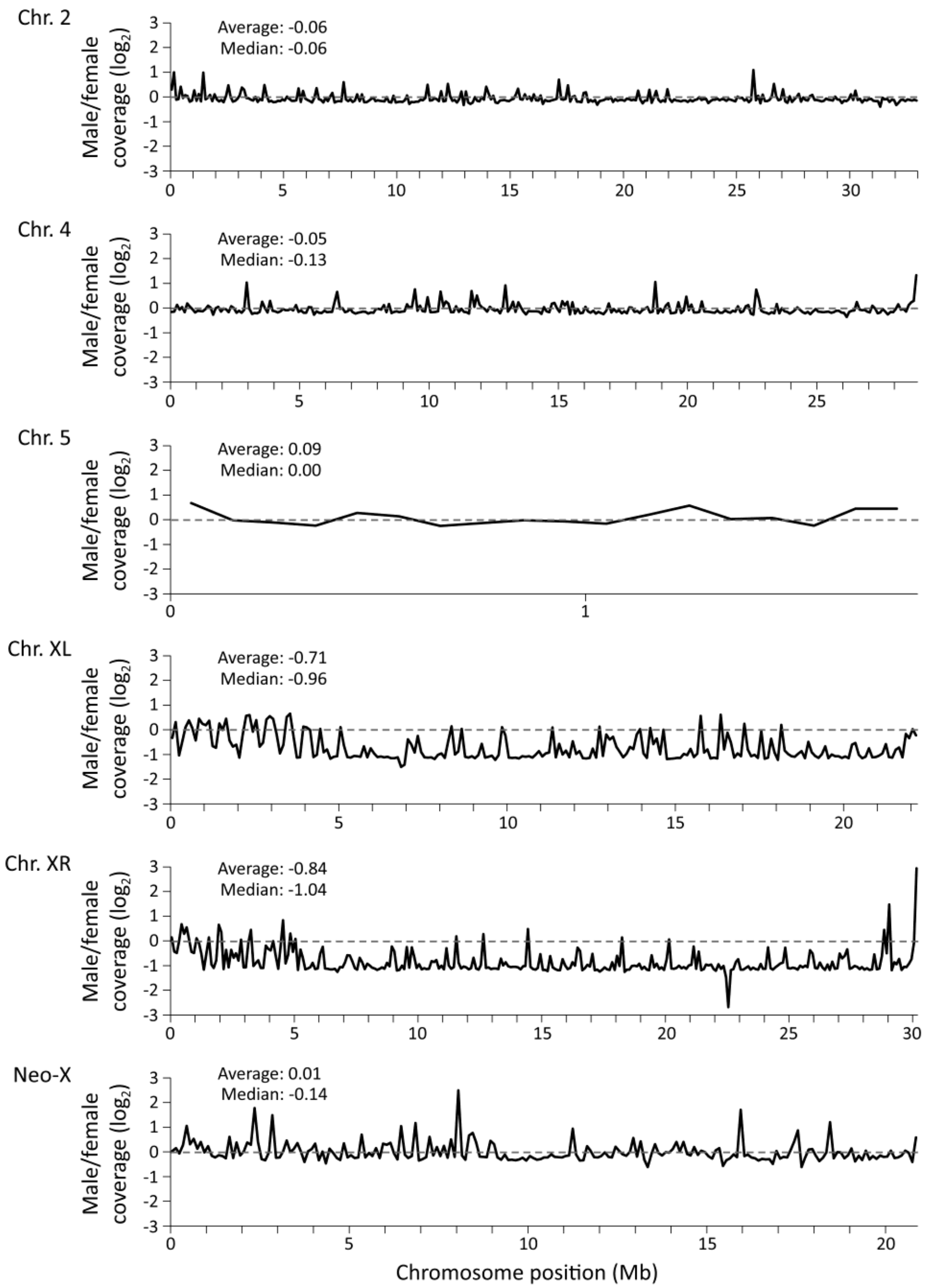

Supplementary Figure 9: Log_2 normalized ratios of male to female read coverage (normalized F/M ratio) on the *D. miranda* chromosome sequences obtained in this study. Window size and sliding size are both 100 kb. In each window, the average F/M ratio was first computed. The ratio was then normalized by dividing by the average F/M ratio (i.e., 1.07) on autosomes. Average and median ratios are shown in margins. Broken lines indicate the equal normalized coverage of males and females.

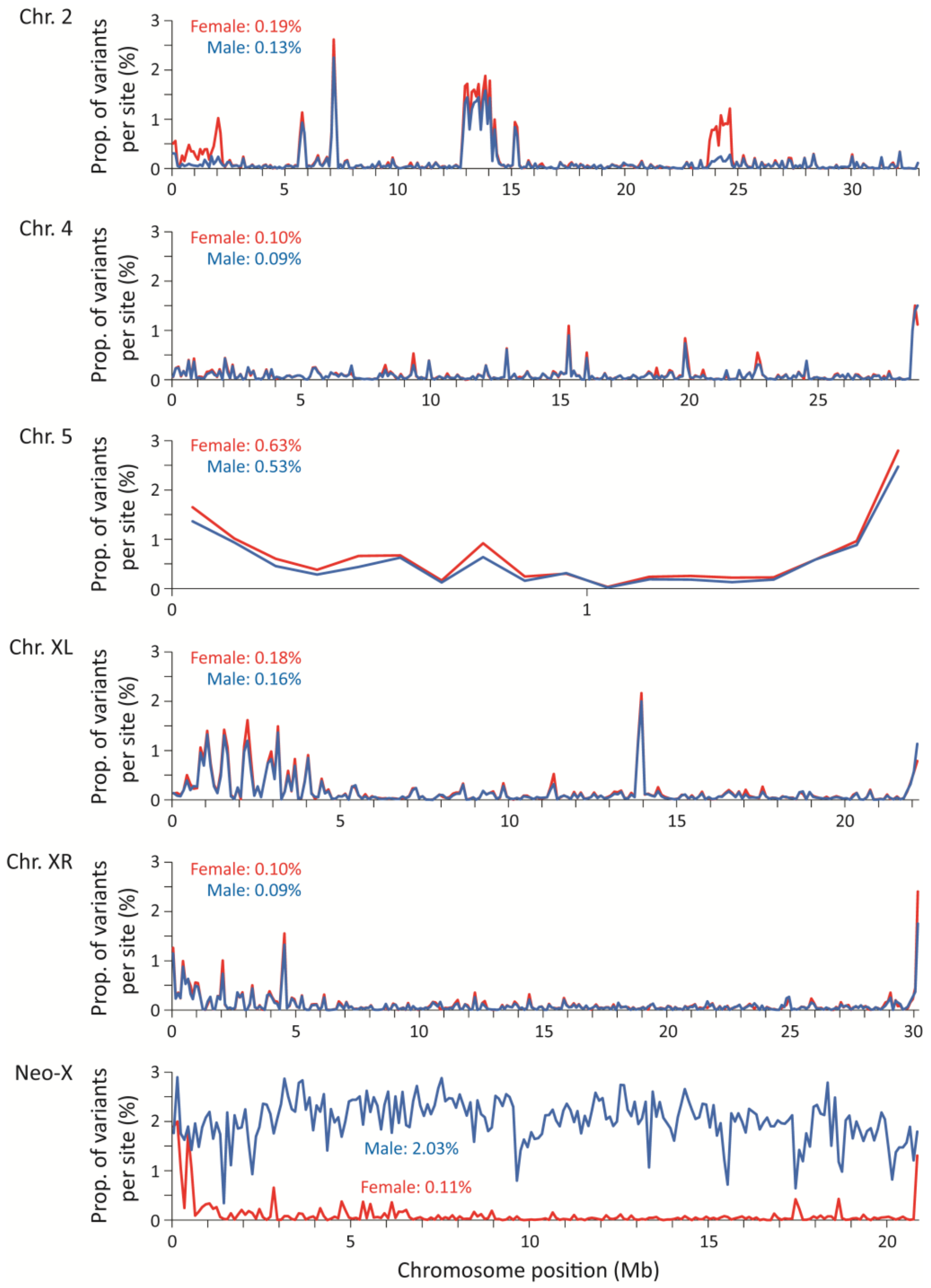

Supplementary Figure 10: Proportion of variants (i.e., SNPs and indels) per site on the *D. miranda* chromosome sequences obtained in this study. Window size and sliding size are both 100 kb. Red and blue lines correspond to female and male values, respectively.

SUPPLEMENTARY TABLES

Supplementary Table 1. Summary of the DNA sequencing data of *Drosophila miranda* and *Drosophila obscura* generated in this study.

Species	Sex	Sample name	Insert size (bp)	Layout/length (bp)	Accession	# read pairs	Purpose
D. miranda	Female	mirF300	~300	Paired end/101	DRR055276	92,306,530	Resequence
	Male	mirM300	~300	Paired end/101	DRR055277	76,123,802	Resequence
D. obscura	Female	obsF300	~300	Paired end/101	DRR055278	228,051,418	*De novo* assembly
		obsF3k	~3k	Mate pair/101	DRR055279	105,714,489	*De novo* assembly
		obsF8k	~8k	Mate pair/101	DRR055280	87,263,335	*De novo* assembly

Supplementary Table 2. Statistics of the *Drosophila miranda* genome (strain 14011-0101.17) in comparison with the reference genome (MSH22).

Chromosome	Strain 14011-0101.17 (this study)		MSH22 (reference genome)	
	Length (bp)	Prop. of N (%)	Length (bp)	Prop. of N (%)
2 (E*)	32,985,154	3.0	33,007,066	2.5
4 (B)	28,806,263	3.6	28,826,359	3.1
5 (F)	1,772,585	11.7	1,772,562	11.4
XL (A)	22,106,965	3.9	22,123,056	3.5
XR (D)	30,111,478	3.1	30,136,903	2.7
Neo-X (C)	20,850,947	3.5	20,862,834	3.2
Neo-Y (C)	20,824,900	3.6	-	-
Total	157,458,292	3.5	136,728,780	3.1

* Muller element

Supplementary Table 3. Statistics of the *Drosophila obscura* genome (strain 14011-0151.01).

Contig	
Max size (bp)	494,872
N50 (bp)	33,766
Average size (bp)	13,796
Number	11,866
Total size (bp)	163,704,451
Scaffold	
Max size (bp)	3,524,470
N50 (bp)	472,512
Average size (bp)	93,988
Number	1,935
Total size (bp)	181,868,570
Prop. of N (%)	4.8

Supplementary Table 4. Summary of the RNA sequencing data of *Drosophila miranda* generated in this study.

Tissue	Sex	Sample name	Layout/length (bp)	Accession	# read pairs	Purpose
Larval whole body	Female	mirLF1	Paired end/101	DRR055176	26,017,355	Transcriptome/gene expression
		mirLF2	Paired end/101	DRR055177	28,918,024	Transcriptome/gene expression
	Male	mirLM1	Paired end/101	DRR055178	25,590,609	Transcriptome/gene expression
		mirLM2	Paired end/101	DRR055179	25,158,302	Transcriptome/gene expression
Pupal whole body	Female	mirPF1	Paired end/101	DRR055186	24,194,242	Transcriptome/gene expression
		mirPF2	Paired end/101	DRR055187	24,271,252	Transcriptome/gene expression
	Male	mirPM1	Paired end/101	DRR055188	27,069,649	Transcriptome/gene expression
		mirPM2	Paired end/101	DRR055189	28,447,437	Transcriptome/gene expression
Head	Female	mirHF1	Paired end/101	DRR055172	24,039,012	Transcriptome/gene expression
		mirHF2	Paired end/101	DRR055173	24,961,270	Transcriptome/gene expression
	Male	mirHM1	Paired end/101	DRR055174	28,023,099	Transcriptome/gene expression
		mirHM2	Paired end/101	DRR055175	31,100,365	Transcriptome/gene expression
Thorax	Female	mirTF1	Paired end/101	DRR055190	25,538,074	Transcriptome/gene expression
		mirTF2	Paired end/101	DRR055191	25,138,928	Transcriptome/gene expression
	Male	mirTM1	Paired end/101	DRR055192	26,154,052	Transcriptome/gene expression
		mirTM2	Paired end/101	DRR055193	29,503,948	Transcriptome/gene expression
Abdomen	Female	mirAF1	Paired end/101	DRR055158	24,828,621	Transcriptome/gene expression
		mirAF2	Paired end/101	DRR055159	27,728,223	Transcriptome/gene expression
	Male	mirAM1	Paired end/101	DRR055160	27,278,371	Transcriptome/gene expression
		mirAM2	Paired end/101	DRR055161	26,365,178	Transcriptome/gene expression
Imaginal disc	Female	mirDF1	Paired end/101	DRR055168	25,764,000	Gene expression
		mirDF2	Paired end/101	DRR055169	28,783,539	Gene expression
	Male	mirDM1	Paired end/101	DRR055170	31,289,310	Gene expression
		mirDM2	Paired end/101	DRR055171	29,915,961	Gene expression

Supplementary Table 4. Continued.

Tissue	Sex	Sample name	Layout/length (bp)	Accession	# read pairs	Purpose
Larva wo disc	Female	mirLdF1	Paired end/101	DRR055180	91,471,126	Gene expression
		mirLdF2	Paired end/101	DRR055181	28,170,791	Gene expression
	Male	mirLdM1	Paired end/101	DRR055182	23,136,121	Gene expression
		mirLdM2	Paired end/101	DRR055183	18,896,167	Gene expression
Adult whole body	Female	mirWF1	Paired end/101	DRR055196	28,575,225	Gene expression
		mirWF2	Paired end/101	DRR055197	30,788,103	Gene expression
	Male	mirWM1	Paired end/101	DRR055198	30,183,796	Gene expression
		mirWM2	Paired end/101	DRR055199	26,069,442	Gene expression
Abdomen wo gonad	Female	mirAgF1	Paired end/101	DRR055164	29,136,961	Gene expression
		mirAgF2	Paired end/101	DRR055165	27,129,225	Gene expression
	Male	mirAgM1	Paired end/101	DRR055166	25,716,313	Gene expression
		mirAgM2	Paired end/101	DRR055167	25,595,405	Gene expression
Testis	Male	mirTe1	Paired end/101	DRR055194	21,471,221	Gene expression
		mirTe2	Paired end/101	DRR055195	25,396,512	Gene expression
Accessory gland	Male	mirAc1	Paired end/101	DRR055162	26,273,389	Gene expression
		mirAc2	Paired end/101	DRR055163	25,715,261	Gene expression
Ovary	Female	mirOv1	Paired end/101	DRR055184	26,405,152	Gene expression
		mirOv2	Paired end/101	DRR055185	26,234,207	Gene expression

Supplementary Table 5. Summary of the RNA sequencing data of *Drosophila pseudoobscura* generated in this study.

Tissue	Sex	Sample name	Layout/length (bp)	Accession	# read pairs	Purpose
Larval whole body	Female	pseLF1	Paired end/100	DRR055252	23,699,626	Transcriptome/gene expression
		pseLF2	Paired end/100	DRR055253	23,636,731	Transcriptome/gene expression
	Male	pseLM1	Paired end/100	DRR055254	27,189,413	Transcriptome/gene expression
		pseLM2	Paired end/100	DRR055255	20,976,896	Transcriptome/gene expression
Pupal whole body	Female	psePF1	Paired end/100	DRR055262	20,506,898	Transcriptome/gene expression
		psePF2	Paired end/100	DRR055263	47,761,070	Transcriptome/gene expression
	Male	psePM1	Paired end/100	DRR055264	19,444,202	Transcriptome/gene expression
		psePM2	Paired end/100	DRR055265	27,884,695	Transcriptome/gene expression
Head	Female	pseHF1	Paired end/100	DRR055248	33,156,619	Transcriptome/gene expression
		pseHF2	Paired end/100	DRR055249	24,300,483	Transcriptome/gene expression
	Male	pseHM1	Paired end/100	DRR055250	15,689,099	Transcriptome/gene expression
		pseHM2	Paired end/100	DRR055251	23,133,869	Transcriptome/gene expression
Thorax	Female	pseTF1	Paired end/100	DRR055266	25,306,962	Transcriptome/gene expression
		pseTF2	Paired end/100	DRR055267	22,806,894	Transcriptome/gene expression
	Male	pseTM1	Paired end/100	DRR055268	30,585,184	Transcriptome/gene expression
		pseTM2	Paired end/100	DRR055269	20,951,142	Transcriptome/gene expression
Abdomen	Female	pseAF1	Paired end/100	DRR055234	22,453,022	Transcriptome/gene expression
		pseAF2	Paired end/100	DRR055235	22,642,014	Transcriptome/gene expression
	Male	pseAM1	Paired end/100	DRR055236	20,130,921	Transcriptome/gene expression
		pseAM2	Paired end/100	DRR055237	21,041,247	Transcriptome/gene expression
Imaginal disc	Female	pseDF1	Paired end/101	DRR055244	27,764,109	Gene expression
		pseDF2	Paired end/101	DRR055245	28,048,524	Gene expression
	Male	pseDM1	Paired end/101	DRR055246	27,691,927	Gene expression
		pseDM2	Paired end/101	DRR055247	28,313,712	Gene expression

Supplementary Table 5. Continued.

Tissue	Sex	Sample name	Layout/length (bp)	Accession	# read pairs	Purpose
Larva wo disc	Female	pseLdF1	Paired end/101	DRR055256	20,973,974	Gene expression
		pseLdF2	Paired end/101	DRR055257	42,074,479	Gene expression
	Male	pseLdM1	Paired end/101	DRR055258	21,853,977	Gene expression
		pseLdM2	Paired end/101	DRR055259	21,719,428	Gene expression
Adult whole body	Female	pseWF1	Paired end/101	DRR055272	25,660,366	Gene expression
		pseWF2	Paired end/101	DRR055273	27,761,070	Gene expression
	Male	pseWM1	Paired end/101	DRR055274	30,144,127	Gene expression
		pseWM2	Paired end/101	DRR055275	25,006,521	Gene expression
Abdomen wo gonad	Female	pseAgF1	Paired end/101	DRR055240	24,174,001	Gene expression
		pseAgF2	Paired end/101	DRR055241	25,140,448	Gene expression
	Male	pseAgM1	Paired end/101	DRR055242	25,236,268	Gene expression
		pseAgM2	Paired end/101	DRR055243	26,230,153	Gene expression
Testis	Male	pseTe1	Paired end/101	DRR055270	28,061,108	Gene expression
		pseTe2	Paired end/101	DRR055271	26,790,630	Gene expression
Accessory gland	Male	pseAc1	Paired end/101	DRR055238	22,949,038	Gene expression
		pseAc2	Paired end/101	DRR055239	28,394,642	Gene expression
Ovary	Female	pseOv1	Paired end/101	DRR055260	26,106,548	Gene expression
		pseOv2	Paired end/101	DRR055261	29,728,159	Gene expression

Supplementary Table 6. Summary of the RNA sequencing data of *Drosophila obscura* generated in this study.

Tissue	Sex	Sample name	Layout/length (bp)	Accession	# read pairs	Purpose
Larval whole body	Female	obsLF1	Paired end/101	DRR055214	24,786,059	Transcriptome/gene expression
		obsLF2	Paired end/101	DRR055215	28,130,180	Transcriptome/gene expression
	Male	obsLM1	Paired end/101	DRR055216	27,068,506	Transcriptome/gene expression
		obsLM2	Paired end/101	DRR055217	25,176,259	Transcriptome/gene expression
Pupal whole body	Female	obsPF1	Paired end/101	DRR055218	20,677,458	Transcriptome/gene expression
		obsPF2	Paired end/101	DRR055219	28,506,170	Transcriptome/gene expression
	Male	obsPM1	Paired end/101	DRR055220	30,742,844	Transcriptome/gene expression
		obsPM2	Paired end/101	DRR055221	25,292,494	Transcriptome/gene expression
Head	Female	obsHF1	Paired end/101	DRR055210	20,653,553	Transcriptome/gene expression
		obsHF2	Paired end/101	DRR055211	25,405,272	Transcriptome/gene expression
	Male	obsHM1	Paired end/101	DRR055212	31,306,138	Transcriptome/gene expression
		obsHM2	Paired end/101	DRR055213	24,891,358	Transcriptome/gene expression
Thorax	Female	obsTF1	Paired end/101	DRR055224	23,654,405	Transcriptome/gene expression
		obsTF2	Paired end/101	DRR055225	30,632,421	Transcriptome/gene expression
	Male	obsTM1	Paired end/101	DRR055226	24,903,146	Transcriptome/gene expression
		obsTM2	Paired end/101	DRR055227	25,891,491	Transcriptome/gene expression
Abdomen	Female	obsAF1	Paired end/101	DRR055200	24,987,319	Transcriptome/gene expression
		obsAF2	Paired end/101	DRR055201	28,438,968	Transcriptome/gene expression
	Male	obsAM1	Paired end/101	DRR055202	23,710,714	Transcriptome/gene expression
		obsAM2	Paired end/101	DRR055203	28,164,700	Transcriptome/gene expression
Adult whole body	Female	obsWF1	Paired end/101	DRR055230	27,369,387	Gene expression
		obsWF2	Paired end/101	DRR055231	29,481,530	Gene expression
	Male	obsWM1	Paired end/101	DRR055232	30,688,771	Gene expression
		obsWM2	Paired end/101	DRR055233	27,006,540	Gene expression

Supplementary Table 6. Continued.

Tissue	Sex	Sample name	Layout/length (bp)	Accession	# read pairs	Purpose
Abdomen wo gonad	Female	obsAgF1	Paired end/101	DRR055206	25,120,703	Gene expression
		obsAgF2	Paired end/101	DRR055207	25,414,599	Gene expression
	Male	obsAgM1	Paired end/101	DRR055208	31,964,971	Gene expression
		obsAgM2	Paired end/101	DRR055209	31,780,062	Gene expression
Testis	Male	obsTe1	Paired end/101	DRR055228	27,562,422	Gene expression
		obsTe2	Paired end/101	DRR055229	22,517,871	Gene expression
Accessory gland	Male	obsAc1	Paired end/101	DRR055204	25,404,520	Gene expression
		obsAc2	Paired end/101	DRR055205	27,995,850	Gene expression
Ovary	Female	obsOv1	Paired end/101	DRR055218	24,626,338	Gene expression
		obsOv2	Paired end/101	DRR055219	23,178,468	Gene expression

Supplementary Table 7. Statistics of the transcriptomes and gene annotations of *Drosophila miranda* and closely related species.

Species	#genes	#transcripts	#expressed protein-coding genes*	#protein-coding transcripts	#non-expressed protein-coding genes†
D. miranda	17,541	30,565	12,896	25,264	1,980
D. pseudoobscura	16,395	29,047	12,003	23,950	1,770
D. obscura	16,135	28,370	11,888	23,494	3,657

Greater numbers in *D. miranda* compared with other two species would be due to inclusion of neo-Y genes/transcripts.

Genes/transcripts of the other two species are based on the genome sequences without the Y chromosome.

* Gene annotations are based on TransDecoder-2.0.1[1].

† Gene annotations are based on Augustus-3.0.3[2].

Supplementary Table 8. Numbers of pseudogenization events on each branch with different thresholds regarding the CDS length.

Threshold*	Branch *X*†	Branch *Y*	Branch *Anc*	Branch *Pse*
10%	217	686	84	213
20%	217	686	84	213
30%	218	688	84	214
40%	218	690	84	215
50%	221	699	84	215
60%	229	714	84	218
70%	235	732	84	224
80%	242	749	84	229
90%	266	767	86	261

* Threshold of the CDS length of a gene. If the CDS length was less than the threshold compared with the average of other orthologous members, the gene was regarded as a "disrupted" gene. Other definitions of pseudogenes were the same.

† Branch names are as follows: *Anc*, the ancestral branch before separating the neo-X and neo-Y; *X*, the neo-X branch; and *Y*, the neo-Y branch. Note that the branch *Pse* here indicates an entire branch of the *D. pseudoobscura* lineage after splitting from *D. miranda*. For other parts of the study, the branch *Pse* means the branch corresponding to the *D. pseudoobscura* lineage after the emergence of the neo-sex chromosomes in *D. miranda*.

Supplementary Table 9. Statistical significance by the χ^2 test under the null hypothesis that the rate of pseudogenization is the same between branches with different thresholds of pseudogenization and different emergence times of the neo-sex chromosomes.

Threshold*	*X* vs. *Y*†		*X* vs. *Pse*		*X* vs. *Anc*		*Y* vs. *Pse*		*Y* vs. *Anc*	
	1 Mya	1.5 Mya	1 Mya	1.5 Mya	1 Mya	1.5 Mya	1 Mya	1.5 Mya	1 Mya	1.5 Mya
10%	6.5×10^{-55}		8.1×10^{-10}	3.2×10^{-3}	1.8×10^{-14}	0.11	3.7×10^{-94}	3.5×10^{-73}	2.3×10^{-104}	1.4×10^{-45}
20%	6.5×10^{-55}		8.1×10^{-10}	3.2×10^{-3}	1.8×10^{-14}	0.11	3.7×10^{-94}	3.5×10^{-73}	2.3×10^{-104}	1.4×10^{-45}
30%	5.8×10^{-55}		7.4×10^{-10}	3.1×10^{-3}	1.3×10^{-14}	0.12	2.4×10^{-94}	2.7×10^{-73}	8.9×10^{-105}	6.8×10^{-46}
40%	2.7×10^{-55}		9.1×10^{-10}	3.5×10^{-3}	1.3×10^{-14}	0.12	1.6×10^{-94}	2.1×10^{-73}	3.4×10^{-105}	3.3×10^{-46}
50%	5.9×10^{-56}		3.8×10^{-10}	2.2×10^{-3}	4.3×10^{-15}	0.15	2.4×10^{-96}	4.4×10^{-75}	4.6×10^{-107}	1.3×10^{-47}
60%	3.4×10^{-56}		6.7×10^{-11}	9.5×10^{-4}	2.5×10^{-16}	0.29	1.0×10^{-98}	4.3×10^{-77}	3.6×10^{-110}	5.6×10^{-50}
70%	1.7×10^{-57}		4.0×10^{-11}	8.5×10^{-4}	2.8×10^{-17}	0.44	5.0×10^{-101}	7.6×10^{-79}	6.4×10^{-114}	7.4×10^{-53}
80%	2.3×10^{-58}		1.8×10^{-11}	5.8×10^{-4}	2.1×10^{-18}	0.65	2.0×10^{-103}	1.1×10^{-80}	1.8×10^{-117}	1.3×10^{-55}
90%	8.8×10^{-55}		1.0×10^{-11}	1.1×10^{-3}	8.5×10^{-22}	0.73	4.0×10^{-100}	1.1×10^{-75}	3.0×10^{-120}	6.5×10^{-57}

* Threshold of the CDS length of a gene. If the CDS length was less than the threshold value compared with the average of other orthologous members, the gene was regarded as a "disrupted" gene. Other definitions of pseudogenes were the same.

† The emergence time of neo-sex chromosomes did not affect the numbers of pseudogenization events on the neo-Y (branch *Y*) or neo-X (branch *X*) lineage so that the *P*-value was identical irrespective of the emergence time of neo-sex chromosomes.

Supplementary Table 10. Enriched GO terms in the functional genes on the neo-X chromosome.

GO term	Description	*P*-value	FDR *q*-value	Enrichment	Category
GO:0006897	Endocytosis	2.35E-04	4.40E-02	1.32	Process
GO:0016192	vesicle-mediated transport	5.21E-05	1.46E-02	1.29	Process
GO:0010604	positive regulation of macromolecule metabolic process	1.72E-04	3.87E-02	1.21	Process
GO:0048523	negative regulation of cellular process	5.90E-06	3.31E-03	1.19	Process
GO:0009893	positive regulation of metabolic process	2.28E-04	4.52E-02	1.19	Process
GO:0043228	non-membrane-bounded organelle	2.68E-04	3.17E-02	1.18	Component
GO:0043232	intracellular non-membrane-bounded organelle	2.68E-04	2.64E-02	1.18	Component
GO:0048522	positive regulation of cellular process	1.93E-05	8.11E-03	1.18	Process
GO:0048519	negative regulation of biological process	1.34E-05	6.43E-03	1.17	Process
GO:0048518	positive regulation of biological process	2.85E-05	9.58E-03	1.17	Process
GO:0009653	anatomical structure morphogenesis	2.62E-04	4.65E-02	1.15	Process
GO:0048869	cellular developmental process	2.28E-05	8.53E-03	1.14	Process
GO:0019222	regulation of metabolic process	2.87E-05	8.78E-03	1.13	Process
GO:0031323	regulation of cellular metabolic process	6.27E-05	1.62E-02	1.13	Process

Supplementary Table 10. Continued.

GO term	Description	*P*-value	FDR q-value	Enrichment	Category
GO:0060255	regulation of macromolecule metabolic process	1.36E-04	3.26E-02	1.13	Process
GO:0080090	regulation of primary metabolic process	2.20E-04	4.64E-02	1.13	Process
GO:0044767	single-organism developmental process	1.41E-06	1.59E-03	1.12	Process
GO:0043229	intracellular organelle	1.36E-05	8.08E-03	1.11	Component
GO:0050794	regulation of cellular process	9.89E-07	1.67E-03	1.11	Process
GO:0032502	developmental process	1.72E-06	1.45E-03	1.11	Process
GO:0043226	Organelle	2.60E-05	7.72E-03	1.10	Component
GO:0043231	intracellular membrane-bounded organelle	3.00E-04	2.55E-02	1.10	Component
GO:0043227	membrane-bounded organelle	3.00E-04	2.23E-02	1.10	Component
GO:0065007	biological regulation	4.99E-07	1.68E-03	1.10	Process
GO:0050789	regulation of biological process	2.29E-06	1.54E-03	1.10	Process
GO:0044464	cell part	3.30E-05	6.52E-03	1.06	Component
GO:0044424	intracellular part	1.96E-04	2.91E-02	1.06	Component

GO terms with FDR *q*-value of less than 0.05 are shown.

Supplementary Table 11. Numbers of variant sites in sequence reads.

Muller element	Chromosome	Common	Female only	Male only
E	2	31,809	29,368	11,317
B	4	18,185	10,011	7,364
F	5	6,326	4,855	2,990
A	XL (old-X)	24,488	15,192	10,952
D	XR (2nd-X)	19,323	12,105	9,359
C	Neo-X (3)	12,828	9,541	410,405 (7,101*)

* The expected number of male-specific variants that are derived from the neo-X by chance. For this estimation, the ratio of male- to female-specific variants on XL and XR [(10,952 + 9,359) / (15,192 + 12,105) = 0.74] was used as a control. When the same ratio was applied to the neo-X, the expected number of male-specific variants derived from the neo-X was 9,541×0.74 = 7,101, which corresponded to only 1.3% among the total number of male-specific variants.

SUPPLEMENTARY METHODS

Flies

Drosophila miranda (strain 14011-0101.17) and *D. obscura* (strain 14011-0151.01) were obtained from *Drosophila* Species Stock Center at UC San Diego (https://stockcenter.ucsd.edu). *D. pseudoobscura* with the known genome sequence [strain k-s12 (or 14011-0121.94)] was obtained from Kyorin-Fly (http://www.shigen.nig.ac.jp/fly/kyorin/cgi-bin/index.cgi). To reduce heterozygosity as much as possible, full-sib mating was repeated for eight, eight, and three generations for *D. miranda*, *D. pseudoobscura*, and *D. obscura*, respectively.

DNA extraction for library construction and sequencing

The *D. miranda* genome sequence (MSH22 strain) was already released[3,4]. However, since we used another strain for this study as mentioned above, we resequenced the genome of our own strain. For *D. obscura*, *de novo* genome sequencing was conducted. For *D. pseudoobscura*, we just used the reference genome sequence.

We extracted total DNA from ten and 30 adult flies of *D. miranda* (females and males separately) and *D. obscura* (females), respectively, using the method developed by Boom et al.[5]. Paired-end libraries (insert size of ~300 bp) were constructed for *D. miranda*, whereas a paired-end library (insert size of ~300 bp) as well as mate-pair libraries (insert size of ~3 kbp and 8 kbp) were made for *D. obscura* by Macrogen (Seoul, South Korea). Paired-end sequencing of 101 bp for each of the libraries was performed by Macrogen with a HiSeq 2000 sequencer (Illumina, San Diego, CA).

RNA extraction for library construction and sequencing

Ovaries, testes, and accessory glands were collected from ~20 adults of ~1 week old after eclosion. Imaginal discs (except for genital discs) and other larval body parts were also separately collected from males or females of ~20 third instar larvae (during wandering before pupation). For other tissues, samples were collected from five (but 20 for heads and ten for thoraxes, abdomens, and abdomens without gonads) females or males of third instar larvae (before wandering), pupae (48–60 h after pupation), and adults (72–96 h after eclosion). Total RNA from each of these tissues was extracted using a standard acid phenol-guanidinium thiocyanate-chloroform extraction method[6] or PureLink RNA Mini Kit (Thermo Fisher Scientific, Waltham, MA). The total RNA was treated with DNase I to digest genomic DNA, and then mRNA was purified from total RNA using the NEBNext Poly(A) mRNA Magnetic Isolation Module (NEB, Ipswich, MA). cDNA libraries were constructed from mRNA using the NEBNext Ultra Directional RNA Library Prep Kit for Illumina (NEB). Paired-end sequencing of 100 or 101 bp was performed by Beijing Genomics Institute (Beijing, China) or Macrogen, respectively, with a HiSeq 2000 sequencer.

Genome assembly

For *D. miranda*, all obtained reads from HiSeq 2000 (Supplementary Table 1) were first processed with Cutadapt version 1.6[7] to remove adapter sequences. The processed reads were further treated with SolexaQA++ v3.1[8] to select pairs of sequences with quality score ≥25 for ≥50 consecutive nucleotides for both forward and reverse reads. The selected pairs of reads were then mapped onto the reference MSH22 genome (DroMir2.2.fa) using bwa-0.7.8[9]. All variants (SNPs and indels) of our strains were called by a standard workflow of Picard-1.115 (http://broadinstitute.github.io/picard/) and GATK3.1.1[10]. To call variants on autosomes, both female and male data were used. Only female data were used to call variants on the neo-X, to

avoid wrong variant calls that may be caused by the mapping of the reads derived from the neo-Y onto the neo-X. Using our original scripts, we finally replaced the reference genome sequence with the variants. It should be mentioned that the nucleotides with no coverage by our reads were replaced with N. To obtain the neo-Y assembly, qualified male reads of our sequencing strain (14011-0101.17) were mapped to the neo-X that was assembled above. The same procedures were then conducted to call variants on the neo-X. Since those variants were expected to be caused by the mapping of the neo-Y-derived reads, we obtained the neo-Y assembly by replacing the neo-X sequence with the male-specific variants. Here too, the nucleotides with no coverage on the neo-X were replaced with N. It should be noted that the ratio of the male to female coverages on the neo-X was similar to those on the autosomes (Supplementary Fig. 9). In addition, when female or male reads were mapped onto the *D. miranda* (strain 14011-0101.17) genome obtained, the frequency of variants against this genome sequence on the neo-X was ~20 times higher in males than in females mostly due to the mapping of the neo-Y-derived reads on the neo-X in males but similar for other chromosomes (Supplementary Fig. 10). Note that some male-specific variants may not be derived from the neo-Y but from the neo-X just by chance (i.e., variants on the neo-X might be present in male samples but absent in female samples). However, when we computed the expected number of male-specific variants derived from the neo-X using the ratio of female- to male-specific variants on XL and XR as a control, it was only 7,101 sites (Supplementary Table 11). Since this number was only 1.3% of the male-specific variants, the majority of the male-specific variants on the neo-X were likely to be derived from the neo-Y. Therefore, our replacement strategy to obtain the neo-Y assembly must be appropriate and work with high accuracy. The detailed statistics of the resequencing of our *D. miranda* strain are shown in Supplementary Table 2. It should also be noted that according to Zhou and Bachtrog[4] and Vicoso and Bachtrog[11] the ratio of the male to

female coverages on the neo-X was a bit lower than those on the autosomes. However, we used a different strain (14011-0101.17) from them (MSH22) and our resequencing-based assembly was based on the latest version of the reference genome (DroMir2.2.fa). Moreover, our mapping coverage (i.e., ~65-fold for each sex; ~130-fold for autosomes, ~97-fold for the neo-X, and ~32-fold for the neo-Y) was much greater than those in previous studies (i.e., ~60, ~45, and ~15-fold read coverage for autosomes, the neo-X, and the neo-Y, respectively in Zhou and Bachtrog[4] and even lower read coverage in Vicoso and Bachtrog[11]). Therefore, this slight difference is possible and acceptable.

For *D. obscura*, based on the reads obtained (Supplementary Table 1), we conducted a *de novo* genome assembly (strain 14011-0151.01) using ALLPATHS-LG[12] with default options. The read depth against the final genome size was ~470×. The assembled genome sequence was further processed with GapCloser v1.12[13] to remove ambiguous nucleotide sites as much as possible. The detailed statistics of the *D. obscura* genome sequence obtained are shown in Supplementary Table 3.

For *D. pseudoobscura*, we used the available genome assembly (dpse-all-chromosome-r3.1.fasta) which was downloaded from FlyBase (http://flybase.org/).

Transcriptome assembly

To assemble the transcriptome sequence for each of the three species, we used female and male RNA-seq data from whole body larvae, whole body pupae, adult heads, thoraxes, and abdomens (Supplementary Tables 4-6). The RNA-seq data from each sample were first processed with Cutadapt version 1.6 and SolexaQA++ v3.1 as mentioned above.

The filtered paired sequences were then mapped onto the genome assembly for each species obtained above using TopHat v2.0.11[14] with default options. The transcriptome for each

tissue was then constructed by Cufflinks v2.2.1[15] with default options. Since the transcripts in different tissues are frequently overlapping, the transcriptomes for these tissues were finally merged using the "cuffmerge" command included in Cufflinks. Here, transcriptomes for all tissues in females and males were used to reconstruct the transcripts on autosomes, whereas those in only females or males were utilized to reconstruct the transcripts on the neo-X or the neo-Y, respectively. The detailed statistics of the transcriptomes for each species are shown in Supplementary Table 7.

Gene annotation

We predicted open reading frames (ORFs) within transcripts using TransDecoder-2.0.1[1] with available proteins in *D. pseudoobscura* [dpse-all-translation-r3.1.fasta retrieved from FlyBase (http://flybase.org/)] as a database and 50 amino acids as a minimum length. We also predicted the ORFs *ab initio* in each genome using Augustus-3.0.3[2] with the fly default option (i.e., --species=fly). If TransDecoder and Augustus predicted ORFs in an overlapping region on a genome, the annotation based on TransDecoder was adopted because TransDecoder was based on the transcriptome data so that it was clear that the region is at least transcribed. In such cases, the annotation based on Trans Decoder would be more reliable than the annotation by Augustus. The detailed statistics of the annotation for each species are shown in Supplementary Table 7. Here, to remove the false identification of genes on the neo-Y as much as possible, we applied the following additional criteria to annotate neo-Y genes. I) A neo-Y gene contains at least one nucleotide differences from its homolog on the neo-X (only when there is an ortholog on the neo-X). II) A ratio of average male coverage to average female coverage of the gene region on the neo-X is greater than 0.69 (corresponding to the bottom 2.5 percentile of the ratio on autosomes). If none of the criteria was satisfied, the genomic regions on the neo-X and the neo-Y

were regarded to be so diverged that the neo-Y reads in males were unable to be mapped on the neo-X. Therefore the gene on the neo-Y was removed from the analyses as a false detection of the neo-Y genes.

Gene classification

All annotated genes above were classified as follows. First, the longest amino-acid sequences for each of the genes were identified and extracted in each species. Using these sequences, a BLASTP-2.2.28+[16] search was conducted, which identified one-to-one orthologs as reciprocal best-hit sequences among species. Each gene was then classified in the following way. I) If a gene was expressed in tissues of larva, pupa, head, thorax, or abdomen, contained an initiation as well as a stop codon, and its CDS length was equal to or greater than 80% of the average of other members of an orthologous group, the gene was regarded as “functional”. II) If a gene was expressed in at least one of the above tissues, contained an initiation and a stop codon, and its CDS length was less than 80% of the average of other orthologous members, the gene was regarded as being “disrupted”. III) If a gene was expressed in none of the tissues and its CDS length was equal to or greater than 80% of the average of other orthologous members, the gene was being “silenced”. IV) If a gene was expressed in none of the tissues and its CDS length was less than 80% of the average of other orthologous members, the gene was regarded as being “silenced and disrupted”. V) If a gene was expressed in at least one of the tissues, lacked either one of an initiation or a stop codon, and its flanking 100 nucleotides did not contain any ambiguous site, the gene was being “disrupted”. VI) If a gene was expressed in at least one of the tissues, lacked either one of an initiation or a stop codon, and flanking 100 nucleotides contained at least one ambiguous site (i.e., N), the gene was regarded as being “unclassified”.

For genes that did not have any orthologs in other species, the following classification

was applied. I) If a gene was expressed in at least one of the tissues and contained both an initiation and a stop codon, the gene was regarded as being “functional”. II) If a gene was expressed in at least one of the tissues, lacked either one of an initiation or a stop codon, and flanking 100 nucleotides did not contain any ambiguous site, the gene was regarded as being “disrupted”. III) If a gene was expressed in none of the tissues, the gene was being “silenced”. IV) If a gene was expressed in at least one of the tissues, lacked either one of an initiation or a stop codon, and flanking 100 nucleotides contained at least one ambiguous site, the gene was regarded as being “unclassified”.

Assignment of pseudogenization events

To compare the rate of pseudogenization in each evolutionary lineage, we analyzed the orthologs that are functional in *D. obscura*, are present in at least one of *D. miranda* and *D. pseudoobscura*, and have not experienced any inter-chromosomal translocation in the lineages of *D. miranda* or *D. pseudoobscura* after splitting from *D. obscura*. (In other words, all these genes were functional in the common ancestor of the three species.) Under a parsimony framework, we assigned the pseudogenization events on each of the lineages after the divergence from *D. obscura*. For example, if an orthologous gene is functional in *D. pseudoobscura* and *D. obscura* but silenced (, disrupted, or deleted) on the neo-X and neo-Y, the pseudogenization is assumed to have occurred in the ancestral lineage of *D. miranda* (i.e., branch *Anc* in Fig. 2B). Yet, if the cause of pseudogenization of the gene is different (e.g., silencing of the gene on the neo-X whereas disruption of the gene on the neo-Y), the pseudogenization was assigned to the both neo-X (branch *X*) and neo-Y (branch *Y*) lineages, independently, but not to the ancestral lineage.

Estimation of evolutionary rates

To compute the evolutionary rate of each ortholog on each of the branches after splitting from *D. pseudoobscura*, we analyzed the 1,282 orthologs which are likely to be functional in *D. obscura* and not to have experienced any inter-chromosomal translocation after the species split from *D. obscura*. We first aligned amino-acid sequences for each of the orthologs using Muscle[17]. The aligned amino-acid sequences were converted to nucleotide sequences with our own script[18]. The numbers of synonymous (d_S) and nonsynonymous (d_N) substitutions per site were estimated using the modified Nei-Gojobori method[19] with a transition/transversion ratio of 2 with our own script[18]. The branch lengths of synonymous and nonsynonymous substitutions were then estimated based on the minimum-evolution method[20].

Estimation of gene expression level

To examine the expression level of each mRNA (only ORF-containing mRNAs were considered) in each of the three species, the RNA-seq data from each sample were first processed with Cutadapt version 1.6 and SolexaQA++ v3.1 as mentioned above.

The selected paired sequences were then mapped onto the mRNA sequences for each species using BLASTN-2.2.28+[16]. For *D. pseudoobscura* and *D. obscura*, all mRNA sequences on all chromosomes (although Y chromosomes were not included) were used. For *D. miranda*, we used all mRNA sequences except those on the neo-Y for females and all mRNA sequences including those on the neo-Y for males. In the homology search, we only considered the pair of reads that were mapped to the same mRNAs with 100% coverage of the reads without any mismatch or gap to avoid mapping the neo-Y reads to the neo-X mRNAs and *vice versa* in *D. miranda* (exactly the same stringency was also applied to other two species). After the mapping, the number of paired reads to each gene was counted. When a pair of reads was mapped to more than one gene with perfect match, the count was equally divided into these genes without any

weighting[21], although such pairs were very minor (<1% in most samples).

The raw counts for each of the genes were then transformed into FPKM (fragments per kilobase of exon per million mapped fragments). When a gene contained multiple transcripts, the longest transcript for the gene was used to compute FPKM. Since we made two biological replicates for each condition (e.g., female larvae), average FPKM for the two replicates was used to represent the expression level of each transcript in each tissue. When two or more samples (e.g., different tissues, different sexes, and different species) were compared, FPKM was converted into the corrected FPKM (cFPKM) by adjusting the median value to 1. The detailed information of each of the RNA-seq runs of *D. miranda*, *D. pseudoobscura*, and *D. obscura* are provided in Supplementary Tables 4, 5, and 6, respectively.

For estimating the gene expression levels in *D. pseudoobscura*, we first estimated cFPKM values for 1,672 orthologous genes that are expressed in at least one of the larval, pupal, head, thorax, and abdomen tissues in *D. pseudoobscura*. cFPKM values less than 0.01 in tissues were converted into 0.01 to avoid infinite ratios in the computation of gene expression ratio of females to males (F/M ratio). If a gene showed the cFPKM values of 0.01 for both females and males in a tissue, we removed the gene from the analysis.

Analysis of sexually antagonistic genes

To examine the relationship between pseudogenization and sexual antagonism, we used the list of candidates of sexually antagonistic genes identified in *D. melanogaster*[22]. Under the assumption that sexually antagonistic genes are the same in *D. melanogaster* and *D. miranda*, we classified the *D. miranda* genes into genes with female-benefit/male-detriment effects, genes with male-benefit/female-detriment effects, genes with no conflict, and unclassified genes based on their best hit homologs in *D. melanogaster* (dmel-all-translation-r6.07.fasta) by using the

BLASTP-2.2.28+[16] search with a cutoff E-value of 10^{-10}.

SUPPLEMENTARY REFERENCES

1. Haas, B.J. *et al.* De novo transcript sequence reconstruction from RNA-seq using the Trinity platform for reference generation and analysis. *Nat Protoc* **8**, 1494-512 (2013).
2. Stanke, M. & Waack, S. Gene prediction with a hidden Markov model and a new intron submodel. *Bioinformatics* **19 Suppl 2**, ii215-25 (2003).
3. Alekseyenko, A.A. *et al.* Conservation and de novo acquisition of dosage compensation on newly evolved sex chromosomes in *Drosophila*. *Genes Dev* **27**, 853-8 (2013).
4. Zhou, Q. & Bachtrog, D. Sex-specific adaptation drives early sex chromosome evolution in *Drosophila*. *Science* **337**, 341-5 (2012).
5. Boom, R. *et al.* Rapid and simple method for purification of nucleic acids. *J Clin Microbiol* **28**, 495-503 (1990).
6. Sambrook, J. & Russell, D.W. *Molecular cloning: a laboratory manual*, (Cold Spring Harbor Laboratory Press, New York, 2001).
7. Martin, M. Cutadapt removes adapter sequences from high-throughput sequencing reads. *EMB.net.journal* **17**, 10-12 (2011).
8. Cox, M.P., Peterson, D.A. & Biggs, P.J. SolexaQA: At-a-glance quality assessment of Illumina second-generation sequencing data. *BMC Bioinformatics* **11**, 485 (2010).
9. Li, H. & Durbin, R. Fast and accurate short read alignment with Burrows-Wheeler transform. *Bioinformatics* **25**, 1754-60 (2009).
10. McKenna, A. *et al.* The Genome Analysis Toolkit: a MapReduce framework for analyzing next-generation DNA sequencing data. *Genome Res* **20**, 1297-303 (2010).
11. Vicoso, B. & Bachtrog, D. Numerous transitions of sex chromosomes in Diptera. *PLoS*

Biol **13**, e1002078 (2015).

12. Gnerre, S. *et al.* High-quality draft assemblies of mammalian genomes from massively parallel sequence data. *Proc Natl Acad Sci USA* **108**, 1513-8 (2011).

13. Luo, R. *et al.* SOAPdenovo2: an empirically improved memory-efficient short-read de novo assembler. *Gigascience* **1**, 18 (2012).

14. Kim, D. *et al.* TopHat2: accurate alignment of transcriptomes in the presence of insertions, deletions and gene fusions. *Genome Biol* **14**, R36 (2013).

15. Trapnell, C. *et al.* Differential gene and transcript expression analysis of RNA-seq experiments with TopHat and Cufflinks. *Nat Protoc* **7**, 562-78 (2012).

16. Camacho, C. *et al.* BLAST+: architecture and applications. *BMC Bioinformatics* **10**, 421 (2009).

17. Edgar, R.C. MUSCLE: multiple sequence alignment with high accuracy and high throughput. *Nucleic Acids Res* **32**, 1792-7 (2004).

18. Nozawa, M., Miura, S. & Nei, M. Origins and evolution of microRNA genes in *Drosophila* species. *Genome Biol Evol* **2**, 180-9 (2010).

19. Zhang, J., Rosenberg, H.F. & Nei, M. Positive Darwinian selection after gene duplication in primate ribonuclease genes. *Proc Natl Acad Sci USA* **95**, 3708-13 (1998).

20. Rzhetsky, A. & Nei, M. Theoretical foundation of the minimum-evolution method of phylogenetic inference. *Mol Biol Evol* **10**, 1073-95 (1993).

21. Nozawa, M., Fukuda, N., Ikeo, K. & Gojobori, T. Tissue- and stage-dependent dosage compensation on the neo-X chromosome in *Drosophila pseudoobscura*. *Mol Biol Evol* **31**, 614-24 (2014).

22. Innocenti, P. & Morrow, E.H. The sexually antagonistic genes of *Drosophila melanogaster*. *PLoS Biol* **8**, e1000335 (2010).
